# G-quadruplexes in the evolution of hepatitis B virus

**DOI:** 10.1093/nar/gkad556

**Published:** 2023-07-03

**Authors:** Václav Brázda, Michaela Dobrovolná, Natália Bohálová, Jean-Louis Mergny

**Affiliations:** Institute of Biophysics of the Czech Academy of Sciences, Brno, Czech Republic; Institute of Biophysics of the Czech Academy of Sciences, Brno, Czech Republic; Faculty of Chemistry, Brno University of Technology, Purkyňova 118, 612 00 Brno, Czech Republic; Institute of Biophysics of the Czech Academy of Sciences, Brno, Czech Republic; Institute of Biophysics of the Czech Academy of Sciences, Brno, Czech Republic; Laboratoire d’Optique et Biosciences (LOB), Ecole Polytechnique, CNRS, INSERM, Institut Polytechnique de Paris, 91120 Palaiseau, France

## Abstract

Hepatitis B virus (HBV) is one of the most dangerous human pathogenic viruses found in all corners of the world. Recent sequencing of ancient HBV viruses revealed that these viruses have accompanied humanity for several millenia. As G-quadruplexes are considered to be potential therapeutic targets in virology, we examined G-quadruplex-forming sequences (PQS) in modern and ancient HBV genomes. Our analyses showed the presence of PQS in all 232 tested HBV genomes, with a total number of 1258 motifs and an average frequency of 1.69 PQS per kbp. Notably, the PQS with the highest G4Hunter score in the reference genome is the most highly conserved. Interestingly, the density of PQS motifs is lower in ancient HBV genomes than in their modern counterparts (1.5 and 1.9/kb, respectively). This modern frequency of 1.90 is very close to the PQS frequency of the human genome (1.93) using identical parameters. This indicates that the PQS content in HBV increased over time to become closer to the PQS frequency in the human genome. No statistically significant differences were found between PQS densities in HBV lineages found in different continents. These results, which constitute the first paleogenomics analysis of G4 propensity, are in agreement with our hypothesis that, for viruses causing chronic infections, their PQS frequencies tend to converge evolutionarily with those of their hosts, as a kind of ‘genetic camouflage’ to both hijack host cell transcriptional regulatory systems and to avoid recognition as foreign material.

## Graphical Abstract

**Graphical Abstract:**
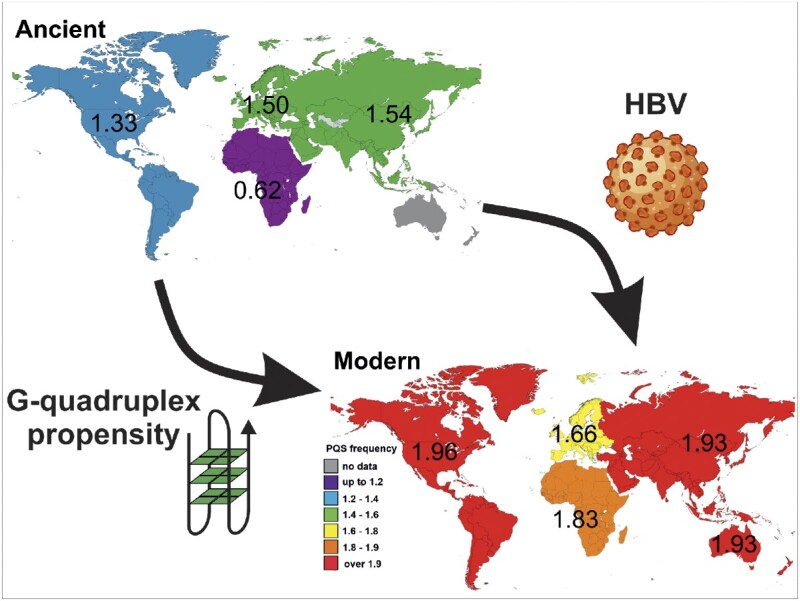
Quadruplex frequencies in ancient (top left) and modern (bottom right) HBV genomes according to the place of discovery (continents).

## INTRODUCTION

Hepatitis B virus (HBV) belongs to the genus Orthohepadnavirus; its genome is constituted of double-stranded DNA. This virus causes Hepatitis B, a highly contagious, potentially fatal disease that affects an estimated 257 million people worldwide, resulting in an estimated 820 000 deaths every year (https://www.who.int/news-room/fact-sheets/detail/hepatitis-b). This virus is particularly severe, as approximately one in five carriers die from cirrhosis and/or develop hepatocellular carcinoma. HBV is transmitted primarily through blood and body fluids and the incubation period is variable, usually between 30 and 180 days. During replication, HBV DNA forms a minichromosome in the nucleus of infected hepatocytes (1,2) and its genome is replicated through a process of reverse transcription of the key intermediate pre-genomic RNA in hepatocytes, which is also an mRNA template for the HBV proteins (3,4). Hepadnaviruses infecting other hosts have recently been identified, including bats, frogs, lizards, fish, and the capuchin monkey (5–8). Analyses of ancient genomes have revealed that the most recent common ancestor of all HBV lineages is estimated to have existed between ∼20 000 and 12 000 years ago, and the virus was found to be present in European and South American hunter-gatherers during the early Holocene period (9).

A large amount of literature is devoted to the occurrence of G-quadruplexes in viruses, especially to the possibility of using these structures in therapy. Comprehensive bioinformatics analyses have traced putative G4-forming sequences in the genome of almost all human viruses, showing that their distribution and presence are highly conserved. Therefore, these DNA or RNA structures can be a suitable target for targeted therapy. Some G-quadruplex ligands have been shown to have antiviral activity, for example, against HIV (10), herpes simplex virus I (HSV-1) (11), SARS-CoV-2 (12) and others. A comprehensive analysis of all sequenced viruses that have a latent phase in their life cycle showed that their G-quadruplex content is correlated with that of the host (13). In contrast, viruses causing acute infections without a latent phase tend to eliminate G-quadruplex sequences, as they can become roadblocks during replication, transcription and/or reverse transcription (14).

More specifically, G4s have been found to be relevant in HBV infection, both at the DNA and RNA level (15). Chakraborty and Ghosh reported that an RNA sequence present in HBV RNA exhibited a sequence-independent trans-acting nuclease activity, and that this sequence adopts a G4 conformation (16). Biswas *et al.* analyzed a G4 prone motif (GGGAGTGGGAGCATTCGGGCCAGGG) that is highly conserved only in HBV genotype B, and was shown to adopt a hybrid structure (17). Interestingly, mutations disrupting this G-quadruplex in HBV genotype B constructs were associated with impaired virion secretion. The authors proposed that this G4 mediates enhancement of transcription and virion secretion in this HBV genotype. In a later review, they note that among viruses containing a G4 in their genome, those associated with cancer are over-represented, including HBV (18).

In contrast, some G4s tend to be conserved in all genotypes, as reported by Meier-Stephenson *et al* (19) for a DNA sequence found in the pre-core promoter region (CTGGGAGGAGCTGGGGGAGGAGA). They demonstrated a role of this quadruplex in viral replication by comparing the wild-type motif to non-G4-forming mutants *in vitro*. Fleming *et al* identified conserved potential G4 sequences in several viral genomes relevant to human health, and showed that these motifs can provide a framework for N6-methyladenosine (m6A) installation within the loops of RNA G4 sequences found in several viruses, including HBV (20). Somkuti *et al.* determined volume changes in three HBV G4 structures using biophysical approaches *in vitro*. They investigated three DNA sequences: GGCTGGGGCTTGGTCATGGGCCATCAG, GGGAGTGGGAGCATTCGGGCCAGGG and TTGGGTGGCTTTGGGGCATGGAC (21). The same group investigated one of these sequences in more details (HepB; GGCTGGGGCTTGGTCATGGGCCATCAG, found in the coding region of the polymerase protein), and analyzed its interaction with G4 ligands *in vitro* (22). Finally, Sun *et al.* recently investigated the role of cellular G4s (motifs found in the host cell genome) in HBV infection, demonstrating that the DDX5 helicase, known to be capable of resolving RNA G4 structures, is a key regulator of the interferon (IFN) response against this virus. DDX5 downregulation is observed during HBV replication and in poor prognosis HBV-related hepatocellular carcinoma (HCC) (23). All of these results point out the links between viral (or host) DNA and RNA G4s and HBV.

In this paper, we have analyzed 232 HBV genomes from samples covering a more than 10-thousand-year history for the presence of G-quadruplex forming sequences. Our results show an evolutionary shift for an increased number of G-quadruplexes in recent HBV viruses, pointing to the importance of G-quadruplexes in the HBV life-cycle in human liver cells.

## MATERIALS AND METHODS

### Genomes

232 HBV alignments were downloaded from the supplementary materials at (9). Sequences were obtained for 122 modern genotypes and different groups of 110 ancient strains, divided into groups based on mPTP classification. As the reference genome, we took NC_003977.2 and, for analyses of phylogenetically related viruses with hosts other than human, we filtered reference genomes from Orthohepadnaviruses. In total, we downloaded 21 additional HBVs having a non-human host, infecting birds (7 genomes), bats (5 genomes), fish (2 genomes), other Mammals (i.e. neither human nor bats; 6 genomes) and amphibians (1 genome, Tibetan frog hepatitis B virus). HBV G4 contents were compared to the gapless human genome, the new telomere-to-telomere assembly of the human genome (24), which was downloaded from NCBI (T2T-CHM13v2.0).

### G4Hunter analyses

All sequences were analyzed using G4Hunter (http://bioinformatics.ibp.cz) to identify PQS sequences. G4Hunter's default parameters were used (25 nucleotides for window size and 1.2 for threshold). These settings have previously been shown to identify experimentally-validated quadruplex structures. The list of all organisms tested and the results of the analyses were downloaded from the supplementary materials at (9).

### Statistical evaluation

Data with G4Hunter results were merged in an Excel file for statistical evaluation. G4Hunter results, lengths, and GC content of analyzed sequences are accessible in Supplementary material 01. A scatter plot was generated in GraphPad Prism (v 8.0.1), Violin plots were constructed in R (v 4.2.0) with ggplot2. Statistical significance was tested using Student's T-test. Normality of data was determined using Shapiro-Wilk test.

### Construction of LOGO sequence

All sequences for ancient and modern HBV genomes were uploaded into UGENE software (25) and the location of PQS sequences were extracted using ClustalW alignment. LOGO sequence was generated in aligned sequences and WebLogo 3 tool (26).

## RESULTS

**Figure 1.**
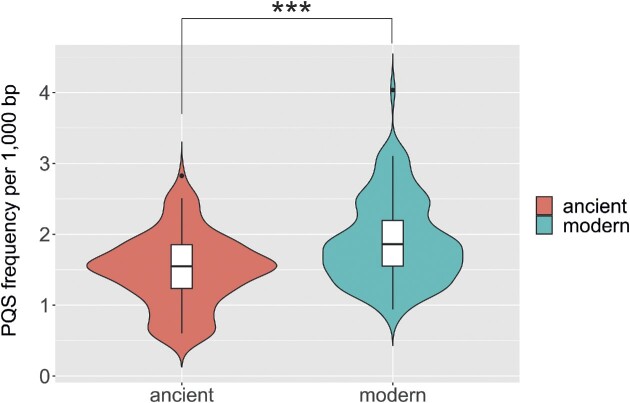
Comparison of PQS frequencies in ancient and modern HBV genomes.

We analyzed the presence of PQS in 232 HBV genomes (122 ancient and 110 recent) using G4Hunter. All human HBV genomes are similar in length, varying from 3180 to 3300 bp. Comparisons of ancient and recent samples show a slight and non-significant change in average length from 3217 to 3210 bp. On the other hand, these genomes vary in GC content, and we found the presence of G-quadruplex-forming sequences in all HBV genomes in the dataset. In total, we found 1258 PQS with a mean frequency of 1.69 PQS per kbp (Table 1). The mean frequency of PQS in ancient genomes is 1.50/kb, compared with the mean frequency in modern HBV genomes of 1.90. For comparison we also analyzed the newly published human gapless assembly. The PQS frequency in the human genome is 1.93, which is almost identical to the average PQS frequency of modern HBV genomes (Table 1). Although there is no significant change in the length of ancient and modern HBV genomes, comparison of PQS density shows that the modern viruses are substantially richer in PQS (Figure 1) (*P*-value = 3.6e-08). The modern HBV genomes not only have a higher PQS frequency, but also have a higher GC content. To evaluate if the change in PQS frequency is statistically significant after taking into account GC content, we recalculated the PQS frequency according to GC content (Table 1, last column). Even after this correction, the PQS frequency/GC content is higher in modern than in ancient HBV genomes (3.88 versus 3.46 per thousand GC for the modern and ancient HBV genomes, respectively: *P*-value = 1.8e-03).

**Table 1.** Statistic data for G4Hunter analyses of HBV viruses. n Seq (number of strains), Length (length of the sequence, nt), GC % (average GC content), PQS n (total number of predicted PQS with a G4Hunter score of 1.2 or more), Mean PQS f (average number of predicted PQS), Min PQS f (lowest frequency of predicted PQS), Max PQS f (highest frequency of predicted PQS), PQS f per 1000 GC (PQS frequency per 1000 GC)

Groups	Seq n	Length nt	GC %	PQS n	Mean PQS	Min PQS	Max PQS	PQS per GC
**All HBV**	232	3214	45.8	1258	1.69	0.61	4.04	3.66
**Groups**	**Seq n**	**Length nt**	**GC %**	**PQS n**	**Mean PQS**	**Min PQS**	**Max PQS**	**PQS per GC**
**ancient**	122	3217	43.1	587	1.50	0.61	2.83	3.46
**modern**	110	3210	48.8	671	1.90	0.94	4.04	3.88
***H. sapiens* T2T**	1*	3.05 × 10^9^	41.6	5.45 10^6^	1.93	-	-	4.72
Subgroups**	**Seq n**	**Length nt**	**GC %**	**PQS n**	**Mean PQS**	**Min PQS**	**Max PQS**	**PQS per GC**
**A**	21	3227	47.0	105	1.55	2.00	8.00	3.30
**B**	14	3222	49.0	120	2.66	1.55	4.04	5.43
**C**	16	3222	49.1	96	1.86	1.55	3.10	3.79
**D**	57	3190	47.7	317	1.74	0.94	2.83	3.66
**E**	3	3218	48.4	25	2.59	2.17	2.80	5.35
**F**	13	3222	48.8	81	1.93	1.24	2.48	3.96
**G**	3	3255	48.2	12	1.23	1.23	1.23	2.55
**H**	5	3222	48.9	39	2.42	1.86	3.11	4.94
**I**	3	3219	48.8	20	2.07	1.55	2.49	4.24
**J**	1	3187	49.0	4	1.26	1.26	1.26	2.56
**Ancient American**	4	3214	38.4	15	1.17	0.93	1.55	3.12
**Early Anatolian farmer**	1	3189	33.7	2	0.63	0.63	0.63	1.86
**Mesolithic**	8	3192	45.8	33	1.29	0.63	2.20	3.20
**WENBA**	65	3229	42.1	288	1.37	0.61	2.20	3.27
**oru**	2	3191	49.1	8	1.25	1.25	1.25	2.56
**gbn**	3	3194	49.1	20	2.09	1.57	2.51	4.25
**czp**	3	3191	48.3	18	1.88	1.25	2.51	3.89
**other**	10	3211	45.2	55	1.71	0.62	4.04	3.81

*Complete telomere-to-telomere human genome (22 + X + Y chromosomes)

**The groups were defined by Kocher *et al.* (10) and is based on the multi-rate Poisson Tree Processes (mPTP) as a results of genetic clusters number considering a phylogenetic input tree (26).

**Figure 2.**
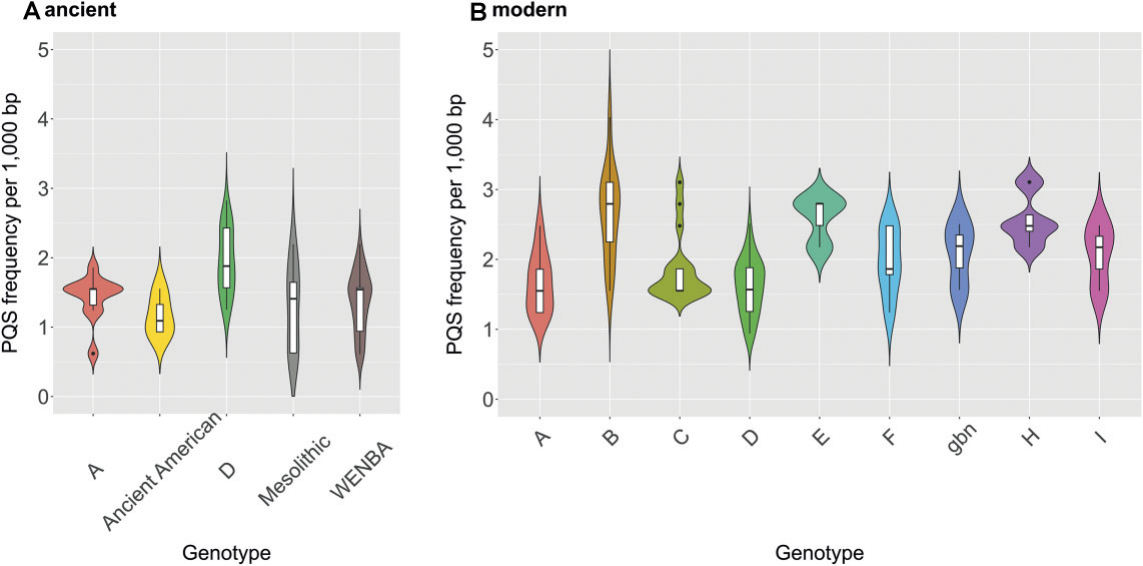
PQS frequency for HBV subgroups divided into ancient and modern genotypes. Ancient American 1, 2, 3 categories were merged. Mesolithic 1 and 2 genotypes were merged.

We then further divided genomes according to different genotypes. While most current genotypes have an average PQS frequency higher than 2 PQS/kb, four of the five ancient genomes have PQS frequencies lower than this value. The highest PQS frequencies were found in modern genotypes B, E and H, the lowest in ancient American, Mesolithic and other ancient genotypes (Figure 2).

We present the PQS frequency per kb (for all motifs with a G4Hunter score >1.2) and the length of the genome as a function of time for each ancient sequence (Supplementary material 02). As mentioned before, the longest sequence only differs from the shortest by 120 bp, and the length of the genome does not change significantly over time (Pearson *r* = −0.1387, *P* (two-tailed) = 1.6e-01). Unlike genome length, PQS frequencies per kbp were found to increase over time (Pearson *r* = 0.2903, *P* (two-tailed) = 2.8e-03; Supplementary material 02).

The whole genome of HBV is transcribed into one long pre-genomic RNA (mRNA-pgRNA) that encodes all HBV proteins, and pgRNA also acts as a template for reverse transcription. Analysis of PQS localization in the HBV reference genome (NC_003977.2) shows that all PQS are located in the vicinity of gene regions, which is not surprising considering that the HBV genome is small and the entire genome is used very effectively to produce the few proteins necessary for its function, such as DNA polymerase, transactivation and capsid proteins (Table 2).

**Table 2.** Position of G4 sequences in the human HBV reference genome (NC_003977.2) and its location conservation. A negative score corresponds to a C-rich sequence, meaning that it is its complementary strand which will be G4-prone

Position	Strand	Sequence	G4H score	Feature/location	G4 conservation
316	−	**CCCC** AA **CC** T **CC** AAT **C** A **C** T **C** A **CC**	−1.41	S, DNA polymerase	
				N-terminal domain	84.5%
737	+	**GG** AT **G** AT **G** T **GG** TATT **GGGGG**	+1.50	S, DNA polymerase	
				C-terminal domain	70.3%
1133	−	**CC** TGAA **CC** TTTA **CCCC** GTTG **CCC**	−1.30	P, DNA polymerase	
				C-terminal domain	19.4%
1722	+	**GGG** A **GG** AGTT **GGGGG** A **GG** A **G** ATTA **GG**	+1.65	Transactivation protein X	92.2%
1887	+	**GGG** T **GG** CTTT **GGGG** CAT **GG**	+1.63	C, Hepatitis core protein	40.5%

**Figure 3.**
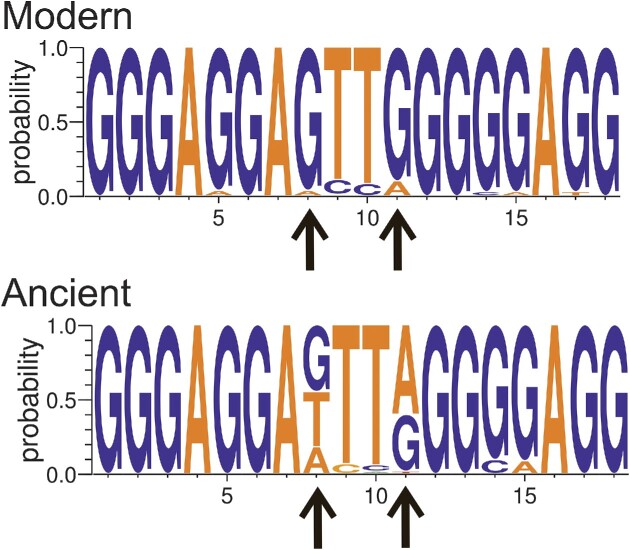
LOGO representation of the consensus motif found around position 1722 (according to the reference HBV genome) in modern and ancient HBV genomes. Guanine nucleotides predominate in modern strains at positions 8 and 11 (arrows) while in ancient HBV genomes other nucleotides are present (T and A in position 8). A less striking G-enrichment is also found at positions 14 and 15. As a consequence, while both consensus motifs are compatible with G4 formation, the modern sequence is more favorable than the ancient one (G4hunter scores of 2.11 and 1.83, respectively).

The PQS with the highest G4Hunter score in the HBV reference genome is located surrounding position 1722, in the region that codes for transactivation protein X. Interestingly, a PQS is present in almost all HBV genomes at this location (214 of 232; or 92.2% of HBV genomes analyzed), making it the most conserved motif between all PQS in the reference HBV genome. Conservation implies that this sequence position has been maintained by selective pressure. Comparison of the LOGO sequence for this location in modern and ancient HBV genomes (Figure 3) demonstrates that a G-rich motif is preserved in all strains. Nevertheless, this ‘G-richness’ is even more striking in modern compared to ancient strains, with Gs becoming predominant at positions 8 and 11 (Figure 3, arrows), while ancient HBV genomes more often exhibit T/A or A at these positions. A higher—near 100%—prevalence of Gs is also visible at positions 14 and 15 (Figure 3) in modern genomes. Overall, while both ancient and modern consensus motifs are G4-prone, the modern sequence is more favorable, as shown by the higher G4Hunter score.

We also divided HBV genomes according to the geographic place of sampling. Most of the ancient HBV samples were found in Europe, only one in Africa and none in Australia. Nonetheless, ancient genomes have a lower PQS frequency than modern HBV genomes, regardless of their continent of origin (Table 3; also see Graphical Abstract).

**Table 3.** G4Hunter score in HBV viruses, grouped by continent and age

Groups	Seq	Length	Mean PQS	Min PQS	Max PQS
**All**	232	3214	1.69	0.61	4.04
**Ancient**	**Seq**	**Length**	**Mean PQS**	**Min PQS**	**Max PQS**
Australia	0	-	-	-	-
America	7	3217	1.33	0.93	3.11
Africa	1	3229	0.62	-	-
Asia	34	3219	1.54	0.61	2.51
Europe	82	3217	1.50	0.61	2.83
**Modern**	**Seq**	**Length**	**Mean PQS**	**Min PQS**	**Max PQS**
Australia	14	3215	1.93	0.94	3.10
America	31	3216	1.96	0.94	3.11
Africa	15	3205	1.83	1.24	2.80
Asia	110	3206	1.93	0.94	4.04
Europe	12	3209	1.66	1.23	2.48

## DISCUSSION

Pathogens evolve in response to human biological changes alongside sociocultural and technological developments (27). Ancient viral genomes provide information on the evolution of viruses over both time and space and provide insight into the changes that may have occurred in virulence and transmissibility (28). Current advanced techniques for isolation of nucleic acids and sequencing have allowed paleogenomic or ‘archeovirology’ investigations. The infamous 1918 ‘Spanish’ influenza pandemic was the source of the first ancient pathogen genome (29) and archeovirology has been growing rapidly since then. Considering that two-thirds of all human pathogens are viruses (30), paleogenetics of viral genomes provides an interesting viewpoint on human history (31).

While several ancient viral genomes are available, the most comprehensive dataset deals with ancient HBV genomes (28). With more than 200 million people suffering from chronic HBV infection, HBV can be considered as a common virus. HBV has a life-cycle that requires its double-stranded DNA genome to reach the host cell nucleus (32), in contrast with RNA viruses such as *influenza* or *SARS-CoV-2* that cause only acute infections and contain RNA genomes that can be replicated and translated in the cytoplasm. Viruses causing acute infections tend to have a low PQS frequency, while G4s tend to be ubiquitous in most organisms.

G4s in the HBV genome are important structures that regulate transcription and virion secretion in HBV genotype B (15,17). In this report, we analyzed the presence of PQS in multiple HBV genomes, from ancient to current HBV strains. Importantly, we found that PQS frequency is higher in recent compared to ancient strains. It was shown previously that the G4 frequency of dsDNA viruses correlates with the PQS frequency of the host, shown for dsDNA viruses infecting Archaea, Bacteria and Eukaryota (33). In agreement with those data, we found that the density of G4 motifs in modern HBV strains (dsDNA viruses that also experience a latent phase) tends to converge to the overall G4 density of the human genome. We propose that mutations which led to ‘PQS integration’ (new G4 motifs within the HBV genome) were evolutionary preferred and fixed in HBV pathogenic strains during evolution. It seems that the opposite process may occur in viruses causing acute infections, as found for SARS-CoV-2, where the PQS frequency is extremely low compared to the PQS frequencies of other coronaviruses (34,35).

Viruses have highly variable genomes and are prone to mutations, in contrast to cellular and especially multicellular organisms, as described repeatedly. This is especially true for RNA viruses, where the mutation rate is several orders of magnitude higher than in DNA-based genomes. A dramatic *decrease* in GC content has been described for several bacterial species (36) and for some plant species with holocentric chromosomes (37). Similarly, a rapid *decrease* in GC content over time was found for some viruses causing acute infections (and without latency connected to nuclear localization), including Nidovirales, influenza genomes and the contemporary SARS-CoV-2 outbreak (38). Comparison of SARS-CoV-2 genomes showed a strong preference for mutations in GC islands and C > U transitions, leading to a decrease in G4 propensity (39,40). As a consequence, the PQS frequency for these viruses causing only acute infections is generally very low (0.03 for SARS-2 (35), 0.56 for influenza H1N1 genomes (41)).

The opposite trend is true for HBV, which exhibits a latent state and maintains its genome in the nucleus: modern HBV genomes have a significantly higher PQS frequency compared to ancient HBV genomes. Our results are in line with a broad study comparing PQS frequencies in viruses with predominantly persistent or acute types of infection (42). According to that study, viruses causing persistent infection are enriched in PQS compared to acutely infectious viruses. Importantly, this observation is also valid within viruses causing hepatitis: HAV (hepatitis A virus - causing acute infection) have a low PQS frequency, while the *Hepadnaviridae* that cause chronic infections (to which human HBV belongs) have a significantly higher PQS frequency (42).

The presence of G4s depends on guanine content in the genome, and one of the possible advantages of a GC-rich genome is to provide additional gene regulation opportunities. In this respect, G4s have been shown to be important for transcription in higher organisms. An increase in GC content has also been documented in plant species that can grow in seasonally cold climates, possibly indicating an advantage of GC-rich DNA during cell freezing, and the genomic adaptations associated with changing GC content are suggested for grass-dominated biomes during the Tertiary period (37). G4s are often overrepresented in the promoter regions of higher eukaryotes and have also been demonstrated to contribute to directed genome editing in nematodes. For viruses experiencing a latent phase in the nucleus, having a similar genome organization as the host is advantageous to both avoid recognition as unusual (foreign) DNA, and to hijack the host regulatory machinery. In addition, the high GC content of HSV DNA is suggested to act as a protective feature against retrotransposon insertion (43).

As AT-rich regions in humans are mostly associated with condensed chromatin (44), the shift to GC-rich viruses could be important for viruses with latent phase to have a better chance of being active in the future. HBV has a latent period, therefore, the evolutionary pressure to increase GC content and PQS presence could be evolutionary favored. In this model, the original (non-human) pre-HBV host could have had a lower G4 frequency – adaptation to the human host may have led to a host-pathogen PQS convergence and a concomitant increase in G4 density in the virus.

## CONCLUSION

We performed the first paleogenomic analysis of G4 propensity, applied here to the Hepatitis B virus. We found that the density of PQS motifs increased over time, as it is higher in modern than ancient HBV genomes. The frequency in modern viruses is now very close to that of the human genome. This study should pave the way to the paleogenomics analysis of G4 sequences (and other similar motifs) in other pathogens. Unfortunately, historical information about viruses genomes is only rarely available: Archeovirology is a nascent field (45), which faces the same obstacles as modern genomics, but with the additional problem of analyzing partially degraded DNA. As noted by the authors (45), HBV is ‘an excellent target for the recovery of ancient sequences due to its relatively stable, partially double-stranded circular DNA genome, its high prevalence in the human population, and prolonged high viremia during chronic infection’. Double-stranded DNA is generally better preserved than single-stranded DNA or RNA. This may explain why HBV is currently the only instance in which sequence data are available for over 100 ancient viruses. In other situations, such as variola virus, only a few ancient genomes are available (there are only 4, 10 and 11 HSV-1, Parvoviruses and VARV ancient sequences available, respectively (28). Analyses of HIV-1 or the 1918 influenza viruses is possible, but only over a limited period of time.

Archeovirology will be useful to identify polymorphisms important for human adaptation to pathogens, and *vice-versa* during the complex virus-host relationships crucial for continued viral prevalence (28). More paleogenomic data will be needed to test the hypothesis that, for viruses causing chronic infections, their PQS frequencies tend to converge evolutionarily with those of their host. In particular, given recent serious outbreaks, we hope that the analysis of ancient viral pathogens will provide critical knowledge about the nature of new viral diseases.

## Supplementary Material

gkad556_Supplemental_Files

## Data Availability

All data are available in the manuscript and supplementary files. HBV sequences were uploaded from the supplementary materials at (9).
