# Supplementary material for: G-quadruplexes in the evolution of hepatitis B virus
Source: Nucleic Acids Res. 2023 Jul 3;51(14):7198–204. doi: 10.1093/nar/gkad556 (PMC10415126; doi:10.1093/nar/gkad556)

**Supplementary material 02**: Length (black) and PQS density (red) of individual HBV genomes and their linear regression (lines) according to their age.
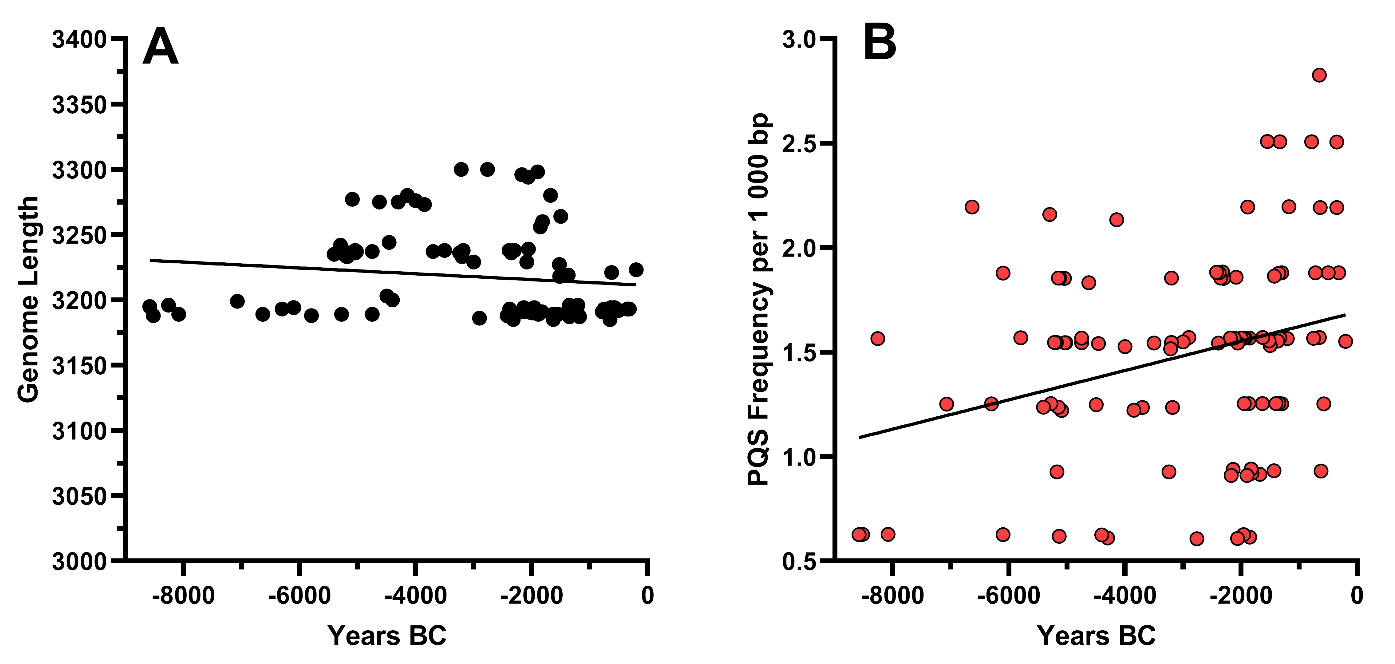

Supplement: gkad556_Supplemental_Files [file gkad556_supplemental_files.zip › SM_02_PQSfreq_.docx]
